# Função Renal e Risco Cardiovascular: Um Aliado Prognóstico Negligenciado

**DOI:** 10.36660/abc.20250414

**Published:** 2025-08-06

**Authors:** Gabriel Porto Soares, Thais Rocha Salim

**Affiliations:** 1 Universidade Federal do Rio de Janeiro Rio de Janeiro RJ Brasil Universidade Federal do Rio de Janeiro, Rio de Janeiro, RJ – Brasil; 2 Universidade de Vassouras Rio de Janeiro RJ Brasil Universidade de Vassouras, Rio de Janeiro, RJ – Brasil

**Keywords:** Função Renal, Risco Cardiovascular, Mortalidade, Síndrome Coronariana Aguda

A subanálise do *Catarina Heart Study* traz evidências relevantes e oportunas para uma relação clínica bem conhecida, porém frequentemente subestimada: o impacto da função renal nos desfechos cardiovasculares. Ao demonstrar que a menor depuração de creatinina na admissão hospitalar prediz, de forma independente, eventos cardiovasculares adversos maiores (MACE) e mortalidade após infarto agudo do miocárdio (IAM), o estudo reforça o rim como um órgão prognóstico essencial em cardiologia.^1^

A interação cardiorrenal não é um conceito novo. A relação bidirecional entre o coração e os rins foi documentada em estudos de larga escala, como o *Chronic Kidney Disease Prognosis Consortium* ^2^ e o ARIC.^3^ O que torna a presente análise relevante é sua aplicabilidade prática e local: em uma população brasileira, utilizando uma ferramenta simples e amplamente disponível — a depuração de creatinina pela fórmula de Cockcroft-Gault — os médicos podem identificar pacientes de alto risco precocemente no curso do IAM.

A força do estudo reside na clareza de sua mensagem: a disfunção renal importa — mesmo em níveis moderados — e importa precocemente. O fato de uma redução modesta na depuração (p. ex., < 60 mL/min) já corresponder a taxas elevadas de MACE corrobora os achados de registros internacionais como o ensaio VALIANT^4^ e o registro GRACE.^5^ Esses estudos, em conjunto, enfatizam que a insuficiência renal não é apenas uma comorbidade, mas um fator contribuinte ativo para o risco cardiovascular.

Em muitos protocolos de síndrome coronariana aguda, a atenção é direcionada para a fração de ejeção, troponinas e cronogramas de reperfusão. Embora estes sejam indubitavelmente essenciais, este estudo exige um reequilíbrio desse foco. A função renal deve ser integrada aos algoritmos de estratificação de risco precoce, principalmente por ser barata, reprodutível e prognosticamente poderosa.^6^

As implicações para a prática clínica são diretas. A identificação de pacientes com função renal comprometida pode refinar decisões sobre estratégias invasivas, uso de contraste, farmacoterapia (como ajuste de dose de antitrombóticos) e intensidade do acompanhamento. Assim, a função renal transcende um rótulo diagnóstico e se torna uma ferramenta de tomada de decisão clínica.^7^

Este editorial também elogia os autores por reconhecerem fatores de risco cardiovascular não tradicionais — como fosfato e ácido úrico — que são influenciados pela insuficiência renal e contribuem ainda mais para danos vasculares. Embora estes não tenham sido medidos no estudo atual, a menção destaca uma visão mais ampla do risco cardiovascular além do colesterol e da pressão arterial.

Existem limitações, como reconhecido pelos autores, particularmente no desenho observacional, na ausência de dados sobre albuminúria e na exclusão de pacientes gravemente enfermos. Ainda assim, o tamanho da amostra, o cenário real e a abordagem estatística robusta (incluindo ajustes de regressão de Cox) conferem credibilidade às conclusões.

É importante ressaltar que o estudo acrescenta dados brasileiros a um campo frequentemente dominado por coortes estadunidenses e europeias. Evidências locais como essas são cruciais para a adaptação de estratégias de saúde pública e para subsidiar diretrizes nacionais.^8^

Em conclusão, a avaliação da função renal — especialmente por meio da depuração da creatinina — não é uma preocupação exclusiva da nefrologia. Na cardiologia, ela precisa ser resgatada como um indicador prognóstico de primeira linha. Este estudo é um chamado à ação para que os médicos revigorem uma ferramenta antiga com nova relevância. O rim pode ser silencioso, mas seu impacto no coração é intenso.

## References

[1] Moreira DM, Sousa MA, Cechinel MFS, Silva RL, Fattah T, Joaquim RM (2025). Association between Renal Function and the Incidence of Major Adverse Cardiovascular Outcomes 1 Year After the First Acute Myocardial Infarction. Arq Bras Cardiol.

[2] Matsushita K, van der Velde M, Astor BC, Woodward M, Levey AS, de Jong PE (2010). Association of Estimated Glomerular Filtration Rate and Albuminuria with All-Cause and Cardiovascular Mortality in General Population Cohorts: A Collaborative Meta-Analysis. Lancet.

[3] Coresh J, Turin TC, Matsushita K, Sang Y, Ballew SH, Appel LJ (2014). Decline in Estimated Glomerular Filtration Rate and Subsequent Risk of End-Stage Renal Disease and Mortality. JAMA.

[4] Smith GL, Lichtman JH, Bracken MB, Shlipak MG, Phillips CO, DiCapua P (2006). Renal Impairment and Outcomes in Heart Failure: Systematic Review and Meta-Analysis. J Am Coll Cardiol.

[5] Fox CS, Muntner P, Chen AY, Alexander KP, Roe MT, Cannon CP (2010). Use of Evidence-Based Therapies in Short-Term Outcomes of ST-Segment Elevation Myocardial Infarction and Non-ST-Segment Elevation Myocardial Infarction in Patients with Chronic Kidney Disease: A Report from the National Cardiovascular Data Acute Coronary Treatment and Intervention Outcomes Network registry. Circulation.

[6] Damman K, Valente MA, Voors AA, O’Connor CM, van Veldhuisen DJ, Hillege HL (2014). Renal Impairment, Worsening Renal Function, and Outcome in Patients with Heart Failure: An Updated Meta-Analysis. Eur Heart J.

[7] Coca SG, Singanamala S, Parikh CR (2012). Chronic Kidney Disease after Acute Kidney Injury: A Systematic Review and Meta-Analysis. Kidney Int.

[8] Ribeiro ALP, Duncan BB, Brant LC, Lotufo PA, Mill JG, Barreto SM (2016). Cardiovascular Health in Brazil: Trends and Perspectives. Circulation.

