# Supplementary material for: Função Renal e Risco Cardiovascular: Um Aliado Prognóstico Negligenciado
Source: Arq Bras Cardiol. 2025 Aug 6;122(7):e20250414. [Article in Portuguese] doi: 10.36660/abc.20250414 (PMC12695033; doi:10.36660/abc.20250414)
Supplement: Translation

# Translation: Renal Function and Cardiovascular Risk: An Overlooked Prognostic Ally

**DOI:** 10.36660/abc.20250414i

**Authors:** Gabriel Porto Soares, Thais Rocha Salim

**Affiliations:** 1 Rio de Janeiro RJ Brazil Universidade Federal do Rio de Janeiro, Rio de Janeiro, RJ – Brazil; 2 Rio de Janeiro RJ Brazil Universidade de Vassouras, Rio de Janeiro, RJ – Brazil

**Keywords:** Renal Function, Cardiovascular Risk, Mortality, Acute Coronary Syndrome

The subanalysis of the *Catarina Heart Study* brings relevant and timely evidence to a well-known yet often underappreciated clinical relationship: the impact of renal function on cardiovascular outcomes. By showing that lower creatinine clearance at hospital admission independently predicts major adverse cardiovascular events (MACE) and mortality after acute myocardial infarction (AMI), the study reinforces the kidney as an essential prognostic organ in cardiology.^1^

Cardiorenal interaction is not a novel concept. The bidirectional relationship between the heart and kidneys has been documented in large-scale studies like the Chronic Kidney Disease Prognosis Consortium^2^and ARIC.^3^What makes the present analysis relevant is its practical and local applicability: in a Brazilian population, using a simple, widely available tool—creatinine clearance by the Cockcroft-Gault formula—clinicians can identify high-risk patients early in the course of AMI.

The study's strength lies in the clarity of its message: renal dysfunction matters—even at moderate levels—and it matters early. The fact that a modest reduction in clearance (e.g., <60 mL/min) already corresponds with elevated MACE rates echoes findings from international registries like the VALIANT trial^4^and the GRACE registry.^5^These studies collectively emphasize that kidney impairment is not merely a comorbidity but an active contributor to cardiovascular risk.

In many acute coronary syndrome protocols, attention is skewed toward ejection fraction, troponins, and reperfusion timelines. While these are undoubtedly essential, this study calls for rebalancing that focus. Renal function should be integrated into early risk stratification algorithms, particularly because it is inexpensive, reproducible, and prognostically powerful.^6^

The implications for clinical practice are direct. Identifying patients with impaired renal function could refine decisions on invasive strategies, contrast use, pharmacotherapy (such as dosage adjustment for antithrombotics), and follow-up intensity. As such, renal function transcends a diagnostic label and becomes a clinical decision-making tool.^7^

This editorial also commends the authors for recognizing non-traditional cardiovascular risk factors—like phosphate and uric acid—that are influenced by renal impairment and further contribute to vascular damage. While these were not measured in the current study, the mention highlights a broader vision of cardiovascular risk beyond cholesterol and blood pressure.

Limitations exist, as acknowledged by the authors, particularly in the observational design, absence of albuminuria data, and exclusion of critically ill patients. Still, the sample size, real-world setting, and robust statistical approach (including Cox regression adjustments) lend credibility to the conclusions.

Importantly, the study adds Brazilian data to a field often dominated by North American and European cohorts. Local evidence like this is crucial for tailoring public health strategies and informing national guidelines.^8^

In conclusion, renal function assessment—especially via creatinine clearance—is not just a nephrology concern. In cardiology, it must be reclaimed as a frontline prognostic indicator. This study is a call to action for clinicians to dust off an old tool with new relevance. The kidney may be silent, but its impact on the heart is loud.
